# Carbon Dioxide Concentrations and Temperatures within Tour Buses under Real-Time Traffic Conditions

**DOI:** 10.1371/journal.pone.0125117

**Published:** 2015-04-29

**Authors:** Chun-Fu Chiu, Ming-Hung Chen, Feng-Hsiang Chang

**Affiliations:** 1 Department of Tourism Affairs, Tzu Hui Institute of Technology, Pingtung, Taiwan (R.O.C.); 2 Department of Tropical Agriculture and International Cooperation, National Pingtung University of Science and Technology, Pingtung, Taiwan (R.O.C.); 3 Department of Recreational Sport and Health Promotion, National Pingtung University of Science and Technology, Pingtung, Taiwan (R.O.C.); 4 Department of Leisure, Recreation and Tourism Management, Tzu Hui Institute of Technology, Pingtung, Taiwan (R.O.C.); Columbia University, UNITED STATES

## Abstract

This study monitored the carbon dioxide (CO_2_) concentrations and temperatures of three 43-seat tour buses with high-passenger capacities in a course of a three-day, two-night school excursion. Results showed that both driver zones and passenger zones of the tour buses achieved maximum CO_2_ concentrations of more than 3000 ppm, and maximum daily average concentrations of 2510.6 and 2646.9 ppm, respectively. The findings confirmed that the CO_2_ concentrations detected in the tour buses exceeded the indoor air quality standard of Taiwan Environmental Protection Administration (8 hr-CO_2_: 1000 ppm) and the air quality guideline of Hong Kong Environmental Protection Department (1 hr-CO_2_: 2500 ppm for Level 1 for buses). Observations also showed that high-capacity tour bus cabins with air conditioning system operating in recirculation mode are severely lacking in air exchange rate, which may negatively impact transportation safety. Moreover, the passenger zones were able to maintain a temperature of between 20 and 25°C during travel, which effectively suppresses the dispersion of volatile organic compounds. Finally, the authors suggest that in the journey, increasing the ventilation frequency of tour bus cabin, which is very beneficial to maintain the travel safety and enhance the quality of travel.

## Introduction

Traffic accidents are the primary cause of injuries and fatalities among tourists during leisure travel [1]. Our review of transportation-related literature shows that the quantity and variance of traffic accident cases are closely related to numerous variables, including climate conditions, socio-economic characteristics, exposure, physical characteristics of the road, safety regulations, and seasonal changes. As such, awareness concerning the significant relationship between tourism and the number of traffic accident cases must be raised [2]. Tour buses are the primary form of transportation for domestic tourism in Taiwan and a key form of public transport due to their high passenger capacity. However, during the occurrence of traffic accidents, tour buses cause greater injury or casualties than other forms of transport, and tour bus passengers are more difficult to rescue [3]. On July 19, 2011, the Consumers’ Foundation, Chinese Taipei published an online poll entitled “Top Ten Transportation Prospects in 2011” that revealed that residents considered “strengthening the control over the lengthy and fatigued driving of public bus drivers” was the primary transportation issue [4]. In actuality, numerous studies have verified that fatigue and sleepiness are the primary causes of traffic accidents [5–7], estimating that 15% to 20% of commercial vehicle casualties are attributed to either fatigue or sleepiness [8,9]. In the past few years, the researchers have examined the concentrations of carbon dioxide (CO_2_) in several types of vehicle cabins and discussed on safety issues because they suspected the elevated CO_2_ concentration leads to drowsiness, lethargy, fatigue, unpleasant feeling at the wheel and limits the ability to concentrate [10–14].

The CO_2_ concentrations in occupied indoor spaces (e.g. tour bus cabins, offices, school classrooms) are higher than concentrations outdoors because humans produce and exhale CO_2_. Prior research has documented direct health effects of CO_2_ on humans (e.g. deepened breathing, respiration increasing markedly, visual disturbances and tremors, loss of consciousness, death), but only at concentrations (20,000–250,000 ppm) much higher than those found in normal indoor settings [15]. Maximum recommended occupational exposure limits for an 8-hr workday are 5,000 ppm as a time-weighted average by the Occupational Safety and Health Administration (OSHA) [16] and the American Conference of Government Industrial Hygienists (ACGIH) [17]. Epidemiologic and intervention research has shown that higher levels of CO_2_ within the range found in normal indoor settings are associated with perceptions of poor air quality, increased prevalence of acute health symptoms (e.g., headache, mucosal irritation), slower work performance, and increased absence [18–23]. Occupants may begin to experience specific health effects at levels in excess of 600 parts per million (ppm). Symptoms may include headache, drowsiness, difficulty concentrating, and dizziness [24]. Other symptoms that may be attributed to high CO_2_ levels include eye irritation, a sensation of stuffy or stale air, and fatigue [25]. It is widely believed that these associations exist only because the higher indoor CO_2_ concentrations at lower outdoor air ventilation rates are correlated with higher levels of other indoor-generated pollutants that directly cause the adverse effects [26,27]. Thus, CO_2_ in the range of concentrations found in buildings (i.e., up to 5,000 ppm) has been assumed to have no direct impacts on occupants’ perceptions, health, or work performance, but evaluated as a common indicator used to assess whether the air exchange rate is sufficient. Nevertheless, recently, the research findings of Satish et al. provided initial evidence (reduce subject’s decision-making performance) for considering CO_2_ as an indoor pollutant, not just a proxy for other pollutants that directly affect people [28].

Previous studies have examined the air quality in various types of vehicle cabins and some of them confirmed that the CO_2_ concentrations in cabins typically exceeded 1000 ppm, which would cause the driver to feel sleepy or fatigued [10–13,29–33]. For example, Chan found that the CO_2_ concentrations aboard a bus in Hong Kong reached 1900 ppm when travelling on the highway and 2500 ppm in the city [29]. Huang and Hsu [31] accessed the CO_2_ concentrations in long-distance bus cabins in Taiwan and reported the hourly average onboard CO_2_ concentration was 959 ppm, ranging from 339 to 3722 ppm. Hsu and Huang [30] monitored the air pollutant concentrations aboard buses travelling long distances on the highway and indicated that the average CO_2_ level is 1463 ppm, which is higher than the guideline for nonindustrial occupied settings [30]. In particular, the results from Hsu and Huang [30] were based on buses carrying between 9 and 19 passengers, for an average of 13.2. In Taiwan, a 43-seat tour bus usually carries between 30 and 40 passengers, suggesting the possibility of even higher CO_2_ concentrations. These results also showed a severe lack of ventilation in commercial buses especially under heavy occupancy conditions, leading to concerns as to whether bus drivers are influenced by the poor air exchange rate and consequently elevating the risk of traffic accidents; besides, the factors that increase the CO_2_ concentrations in vehicle cabins include mainly more passengers onboard [11–13,30], lower driving speed [12,13,30], and setting the air conditioning (AC) system to recirculation mode [12–14]. Likewise, if increased onboard CO_2_ concentrations result in sleepiness and fatigue, then the safety of a tour bus will be jeopardized.

To our knowledge, few studies pertaining to the continuous monitoring of CO_2_ concentrations and temperature changes within high-passenger occupied tour buses with AC system operating in recirculation mode under real-time traffic conditions are currently available. In some previous related studies, buses were operated in the city area [12,13,29,32,33] or on the highway [12,13,30,31] but this study’s buses were performed on a real-life travel tour and went through different road conditions. In addition, some studies carried out only a few or a dozen passengers in vehicles [10,12,13,30] and some involved no real passenger even simulations only [11,14]. Therefore, the present study endeavored to collect CO_2_ concentration and temperature data for 43-seat tour buses to serve as a reference for relevant authorities and vendors to improve travel safety.

## Methods

### Monitoring equipment

Air quality data was recorded using the Smart eHome Wireless Indoor Air Quality (IAQ) Monitoring System, a product of JS Environmental Technology and Energy Saving Co., Ltd. This system uses a non-dispersive infrared sensor (NDIR) to detect CO_2_ concentrations in the air. The core NIDR sensor used to analyze CO_2_, manufactured by a listed Taiwanese optical module manufacturer, is able to detect concentrations within a range of 0 to 3000 ppm. The sensor is able to achieve superior accuracy and stability, demonstrating an accuracy of within ±50 ppm, a reproducibility of within ±20 ppm, and a resolution of 1 ppm, all beneficial for prolonged testing. In addition, this system complies with indoor air quality (CO_2_) monitoring standards, “Methods for Detecting Atmospheric CO_2_—Infrared Sensory,” proposed by the Taiwan Environmental Protection Administration (Taiwan EPA) on 30 January 2013 (NIEA A448.11C) [34].

The IAQ monitoring system employed the ZigBee wireless transmission technology, which enables the system to directly and wirelessly transfer data to a computer over a distance of 50 m (visible distance). This system not only eliminates the need to install sampling tubes and data transmission cables, but also supports Microsoft Windows operating systems and allows users to view tables and charts (instant measurements, minute averages, historical data, and data exports) via internet browsers.

### System quality assessment and control

The system was calibrated by the Industrial Technology Research Institute (ITRI; Green Energy Department). A calibration gas analyzer was employed to perform multi-point calibration at 400, 700, 900, and 1200 ppm test concentrations. The average offset value was between -1.39% and 2.75%, and the linear correlation coefficient was 0.99960 (> 0.995).

Precision inspection was further conducted by simultaneously testing ten identical systems for 24 h. Results showed percentage differences of between -4.2% and 4.8% (within ±10%), with a total coefficient of variation of 2.8%.

The experimental results of the calibration and the precision inspection proved the monitoring system met the control criteria of Method NIEA A448.11C.

### Monitoring strategy

The monitoring system was deployed in three SCANIA 43-seat tour buses manufactured from May 2011 to March 2013. These tour buses were commissioned by a college for a three-day, two-night educational excursion. The system automatically and continuously monitored the CO_2_ concentrations in real time at 1-minute intervals within the tour buses. In addition, the researchers recorded the travelling conditions, opening and closing of the bus doors, and the number of passengers throughout the entire journey.

The system monitored the CO_2_ concentrations and temperature conditions within the tour buses. During the monitoring process, a sensor was placed beside the dashboard and at the center of the emergency door in the passenger section. The sensors were placed at the approximate height of the seated passengers. A wireless transmitter was used to collect data.

### Data analysis

Descriptive statistics were calculated for all three tour buses. The means of the CO_2_ concentrations were calculated separately for each tour day. When calculating and presenting the CO_2_ concentrations, we defined “daily average concentration” as “means of CO_2_ concentrations when the bus departed from the hotel accommodation and ended up finishing the day’s tour.” The minimum recorded value and maximum recorded value for the CO_2_ concentrations were also determined. Finally, the percentage of measured CO_2_ concentrations that exceeded the standard set by the Taiwan EPA for indoor air quality of 1000 ppm was calculated [35].

## Results

**Fig 1:**
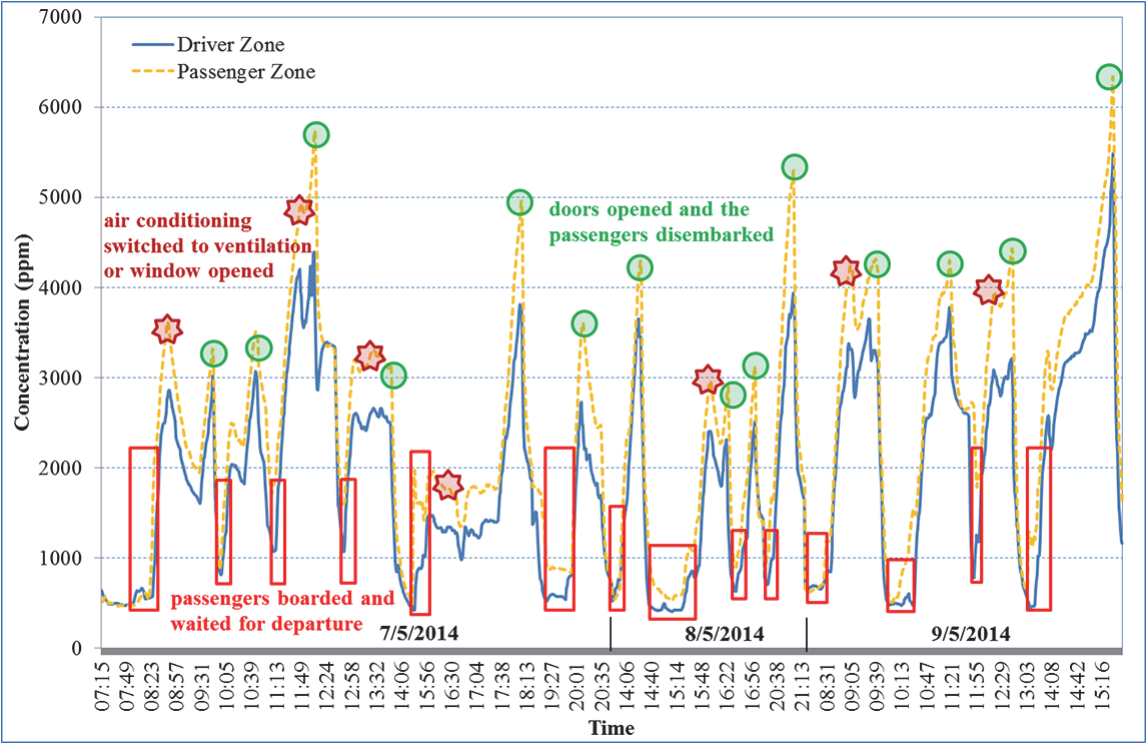
CO_2_ concentrations in the tour bus A as a function of time.

**Fig 2:**
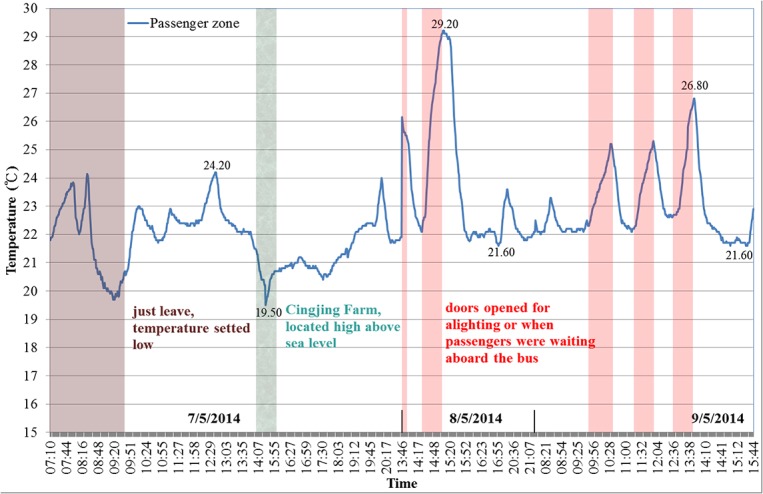
Air temperature in the tour bus A as a function of time.

The CO_2_ concentrations and temperatures within the tour buses were monitored continually from May 7–9, 2014. During the monitoring period, the driver zone comprised one driver and one tour guide, and the passenger zone comprised 40, 41, and 42 students and one teacher each bus for tour bus A, B, and C, respectively. The detection results are listed in Tables 1–3 and illustrated in Figs 1 and 2.

**Table 1:** Results of CO_2_ concentration monitored in tour bus A under real-time traffic conditions.

Tour bus A	Minimum (ppm)	Maximum (ppm)	Mean (ppm)	Percentage of values > 1000 ppm (%)
**Driver Zone**				
**Day 1**	420.0	>3000	1712.2	74.8%
**Day 2**	408.5	>3000	1438.9	56.3%
**Day 3**	458.0	>3000	2102.1	74.5%
**All 3 days**	408.5	>3000	1781.7	71.2%
**Passenger Zone**				
**Day 1**	465.5	>3000	2054.2	80.8%
**Day 2**	524.0	>3000	1760.8	73.4%
**Day 3**	512.5	>3000	2338.3	84.7%
**All 3 days**	465.5	>3000	2087.0	80.6%

Note: for the calculations of the mean CO_2_ concentrations, concentration values above 3000 were set to 3000 because of the upper detection limit of the CO_2_ monitor device was 3000 ppm.

**Table 2:** Results of CO_2_ concentration monitored in tour bus B under real-time traffic conditions.

Tour bus B	Minimum (ppm)	Maximum (ppm)	Mean (ppm)	Percentage of values > 1000 ppm (%)
**Driver Zone**				
**Day 1**	558.0	>3000	2205.2	96.4%
**Day 2**	395.0	>3000	1194.9	38.3%
**Day 3**	456.0	>3000	2093.2	71.1%
**All 3 days**	395.0	>3000	1818.0	68.8%
**Passenger Zone**				
**Day 1**	753.0	>3000	2466.5	97.6%
**Day 2**	400.0	>3000	1207.5	42.3%
**Day 3**	487.5	>3000	2193.4	75.9%
**All 3 days**	400.0	>3000	1943.3	71.9%

Note: for the calculations of the mean CO_2_ concentrations, concentration values above 3000 were set to 3000 because of the upper detection limit of the CO_2_ monitor device was 3000 ppm.

**Table 3:** Results of CO_2_ concentration monitored in tour bus C under real-time traffic conditions.

Tour bus C	Minimum (ppm)	Maximum (ppm)	Mean (ppm)	Percentage of values > 1000 ppm (%)
**Driver Zone**				
**Day 1**	415.0	>3000	2510.6	90.0%
**Day 2**	410.0	>3000	1056.1	31.9%
**Day 3**	503.0	>3000	2181.6	74.1%
**All 3 days**	410.0	>3000	1893.7	64.5%
**Passenger Zone**				
**Day 1**	929.0	>3000	2646.9	98.9%
**Day 2**	464.0	>3000	1381.9	51.6%
**Day 3**	570.5	>3000	2362.2	80.3%
**All 3 days**	464.0	>3000	2111.1	76.5%

Note: for the calculations of the mean CO_2_ concentrations, concentration values above 3000 were set to 3000 because of the upper detection limit of the CO_2_ monitor device was 3000 ppm.

### CO_2_ concentrations in the tour buses

Tables 1–3 list the minimum, maximum, mean, and percentage over 1000 ppm for CO_2_ minute average concentrations within the monitoring period in the driver and passenger zones of the three tour buses A, B, C. The values above 3000 were set to 3000 because of the upper detection limit of the CO_2_ monitor device was 3000 ppm.

For the driver zones, during the three monitoring days, the minimum CO_2_ concentrations were 408.5, 395.0, and 410.0 ppm, the maximum concentration were all >3000 ppm, and the daily average concentration ranged 1712.2–2102.1, 1194.9–2205.2, and 1056.1–2510.6 ppm from each of the three buses A, B, and C. The percentages of the minute average CO_2_ concentrations over 1000 ppm for each day were 74.8%, 56.3%, and 74.5%, for a combined average of 71.2% in bus A; 96.4%, 38.3%, and 71.1%, for a combined average of 68.8% in bus B; 90.0%, 31.9%, and 74.1%, for a combined average of 64.5% in bus C.

For the passenger zone, the minimum CO_2_ concentrations were 465.5, 400.0, and 464.0 ppm, the maximum concentrations were all >3000 ppm, and the daily average concentrations ranged 1760.8–2338.3, 1207.5–2466.5, and 1381.9–2646.9 ppm from each of the three buses A, B, and C. The percentages of the minute average CO_2_ concentrations over 1000 ppm for each day were 80.8%, 73.4%, and 84.7%, for a combined average of 80.6% in bus A; 97.6%, 42.3%, and 75.9%, for a combined average of 71.9% in bus B; 98.9%, 51.6%, and 80.3%, for a combined average of 76.5% in bus C.

The maximum CO_2_ concentrations within the tour buses were all above 3000 ppm (both driver and passenger zones). Passenger zones (daily mean CO_2_ concentrations ranging from 1207.5 to 2646.9 ppm) had higher concentrations of CO_2_ than the driver zones (daily mean concentrations ranging from 1056.1 to 2510.6 ppm). This was largely due to the greater number of passengers in the passenger zones. In addition, the drivers occasionally opened their window. During the three monitoring days, drivers and passengers onboard the tour buses were exposed to CO_2_ concentrations greater than 1000 ppm at least 70% of the time except Day 2.

Fig 1 shows the CO_2_ concentration variations in the driver and passenger zones of the bus A. For buses B and C, the results of the CO_2_ concentration variations were similar. The CO_2_ concentrations in tour bus A gradually escalated from the time passengers boarded the bus and waited for departure until the carriage was fully seated. After all passengers were seated and the bus door closed, the CO_2_ concentrations in the driver zone increased rapidly, increasing at an average rate of approximately 132.4 ppm/min, that was increasing 660 ppm within 5 minutes on average (75.1–228.0 ppm/min; calculated by the authors and data not shown here). When the bus doors opened and the passengers disembarked, the CO_2_ concentrations in the driver zone drastically decreased, decreasing at an average rate of approximately 249.7 ppm/min, that was decreasing 500 ppm within 2 minutes on average (92.0–468.8 ppm/min).

The results suggest that the CO_2_ concentration aboard the bus exceeded 1000 ppm after five minutes of travel. The rate at which CO_2_ decreased was 1.9 times faster than the rate at which it increased. This accelerated reduction was possibly due to the rapid alighting of all the passengers from the front and rear exits of the bus as opposed to the gradual boarding of passengers. In addition, the driver occasionally switched from air conditioning to ventilation during travel, or opened a window, contributing to the reduction of CO_2_ concentrations.

### Temperatures in the tour bus

Fig 2 shows the temperature variations in the passenger zone of the tour bus A. For buses B and C, the results of the temperature variations were similar. The minimum daily temperature in the passenger zone was 19.5°C (19.5–21.6°C), the maximum temperature was 29.2°C (24.2–29.2°C), and the average temperature was 22.6°C (21.8–23.7°C).

The results suggest that the passenger zone maintained a temperature between 20 and 25°C with a lower temperature on the first day. Temperatures exceeding 25°C only occurred when the bus doors opened for alighting or when passengers were waiting aboard the bus. Temperatures below 20°C only occurred at the Cingjing Farm, which was located high above sea level.

## Discussion and Conclusion

Taiwan, located within the subtropical and the tropical climate zones, has warm and mild weather all year round. In 2014, Taipei (located in northern Taiwan) and Kaohsiung (located in southern Taiwan) districts' monthly average temperatures ranged 16.3–30.5°C and 19.5–30.3°C, and annual average temperatures were 23.4°C and 25.6°C, respectively [36]. Therefore, drivers in Taiwan almost all use AC system to keep comfort temperature. However, using the AC system may reduce mileage by 5–25% [37], thus the need to reduce energy consumption provides an incentive for low rates of ventilation, leading to higher indoor CO_2_ concentrations [28]. Although opening windows can help reduce the CO_2_ concentrations in vehicle cabins, drivers tend to use AC system and often set it to recirculation mode to avoid inhaling outside vehicle emissions and to save energy.

In the present study, we examined the CO_2_ concentrations and temperatures of three 43-seat tour buses under real-time traffic conditions. The tour buses were commissioned by a college school for a three-day, two-night educational excursion, and seated 41, 42, and 43 passengers, respectively. This study was different from past research in that it neither examined a public bus traveling on a fixed route nor estimated the CO_2_ concentrations in an experimental chamber or by numerical simulations; it was undertaken in “real” driving scenarios.

Findings from this study revealed that in a relatively short period of time the CO_2_ in the driver zones reached the maximum monitoring concentrations for the equipment used in this study (i.e., 3000 ppm). The CO_2_ concentrations of the passenger zones were even higher than those of the driver zones, achieving an maximum daily average CO_2_ concentration of 2646.9 ppm, indicating that indoor CO_2_ concentrations in tour buses exceed the indoor air quality standard of Taiwan EPA (8 hr-CO_2_: 1000 ppm) [35] and the air quality guideline of Hong Kong Environmental Protection Department (1 hr-CO_2_: 2500 ppm for Level 1 for buses) [38]. The Taiwan EPA announced the Indoor Air Quality Act on November 23, 2011, stipulating that public transportation must be regulated as indoor spaces. However, the list of indoor spaces regulated by this act has not been announced in its entirety; currently, the Taiwan EPA has only announced the first group of indoor spaces (formulated on January 23, 2014 and effective July 1, 2014) which does not include vehicle cabins. If the CO_2_ concentrations in tour bus cabins are not reduced, bus owners may be penalized in the future pursuant to this act.

The CO_2_ concentrations in the driver zones increased 660 ppm on average in first 5 minutes of driving with 43 occupants in the tour bus and decreased 500 ppm on average within 2 minutes when the bus doors opened and the passengers disembarked (Fig 1). The observed sharp rise of CO_2_ (ca. 132.4 ppm/min), which was caused by breathing of the passengers inside the tour bus, is comparable with findings from prior research [10–12]. ČORŇÁK and BRAUN [10] monitored the CO_2_ concentrations in a low middle class car with two occupants on board and found that when the air throttle was adjusted to the position of internal air circulation in the vehicle, the CO_2_ concentrations increased from 450 ppm to 1200 ppm within approximately 6 minutes (i.e., ca. 125 ppm/min). ČORŇÁK et al. [11], used a mathematical model to assess CO_2_ generation inside military vehicles and found that, starting from an initial CO_2_ concentration of 450 ppm, the hygienic limit (1200 ppm) was achieved in 1070 seconds (i.e., ca. 42 ppm/min) with one person in the vehicle, 360 seconds (i.e., 125 ppm/min) for three people, and 120 seconds (i.e., 375 ppm/min) for nine people. Results of Mathur’s research showed that the cabin CO_2_ concentrations reached 1000 ppm in first 5 minutes of driving with only 1 occupant in the vehicle [12]. Based on the above data, it is clear that operation of the vehicle beyond 5 minutes can result in cabin CO_2_ concentration greater than 1000 ppm in some cases. This situation would be much severe if a number of people are sitting inside the vehicle and are going on a long drive.

Our results showed that the CO_2_ concentrations in tour bus cabins are significantly influenced by the number of passengers onboard, which were consistent with those proposed by Mathur [12], Hsu and Huang [30], and Huang and Hsu [31]. The numbers of passengers on the buses A, B, and C (excluding the driver and tour guide) were maintained at 41, 42, and 43, respectively; the corresponding maximum daily average CO_2_ concentrations obtained in the driver zones were 2102.1, 2205.2, and 2510.6 ppm, respectively. Mathur tested a 2003 MY production vehicle driving on highways and reported the peak CO_2_ concentrations in cabin with one and four occupants resulted in 1020 and 2800 ppm [12]. Hsu and Huang [30], and Huang and Hsu [31] both investigated the indoor air quality in long-distance buses in Taiwan. Hsu and Huang indicated the average CO_2_ level in cabins occupied by 9–19 passengers is 1463 ppm [30]. Similarly, Huang and Hsu pointed out in-cabin CO_2_ concentrations were almost all below 1500 ppm and positively associated with the number of passenger (2–23 passengers) [31]. Obviously, the elevated CO_2_ concentrations in tour bus cabins need to be paid more attention due to the high passenger capacity.

The influences of driving speed on cabin CO_2_ concentrations have been discussed by previous studies [29,30]. Chan measured average concentrations of 2513 ppm and 1879 ppm for buses running in city areas and for buses running on highways in Hong Kong [29]. Another similar study by Hsu and Huang [30] indicated that the average CO_2_ concentration measured in the long-distance buses running in city areas was 1683 ppm while on highways was 1463 ppm. The lower CO_2_ levels for the buses on highways are in agreement with a previous study by Mathur [12]. Mathur found that at low vehicle speeds (average 20–25 mph), the magnitudes of the peak cabin concentration for CO_2_ are higher in comparison to higher average vehicle speeds (65 mph). This is due to the fact that at lower vehicle speed there is less air exchange between the inside and outside of the car. Indoor-outdoor air exchange is increased at higher speeds, which helps in reducing cabin CO_2_ concentration. However, our study monitored in-cabin CO_2_ concentrations under complex traffic conditions and was hard to assess the effects of driving speed on CO_2_ concentrations within tour buses, although such effects are conceivable.

Because the driver occasionally switched AC system from recirculation to outside air mode or opened the window beside him during driving, we inadvertently observed that CO_2_ concentrations could be effectively reduced by changing the air circulation in the bus from air conditioning to ventilation, which was not within the experimental scope of this study. These observations were similar to those reported by prior researches [10,12,13,33]. ČORŇÁK and BRAUN [10] suggested that for keeping good air quality in a vehicle (without CO_2_) the air ventilation throttle should be closed for a limited period of time only (e.g., when entering tunnels, driving in heavy traffic in cities, etc.). Mathur [12] recommended if the quality of the outside air is poor then AC system can be changed from outside air mode to recirculation mode. Once the vehicle is out of the traffic jam, the mode door could be switched back to outside air mode. It’s better not to use AC system more than 10 minutes under recirculation mode. Park et al. [13] pointed out the CO_2_ concentration in the interior of a car is increasing over time when in recirculation mode and remains constant during fresh mode; the window open/close effect showed similar results to the AC operation mode. Zhu et al. [33] examined the micro-environmental conditions in public transportation buses and monitored the passengers’ exposures to a variety of environmental conditions in “real world” field campaigns using the Harvard University shuttle bus system. A typical route consisted of four stops and takes approximately 15 minutes. The buses were air-conditioned, and all of their windows were fully closed when in operation. During four full day field campaigns, the average CO_2_ concentrations ranged 678–1025 ppm, which were much lower than that of our study because the bus doors being opened and closed frequently. However, the present study verified that high-capacity tour bus cabins with AC system operating in recirculation mode are severely lacking in air exchange rate.

In addition, bus cabins are specifically constructed environments. Volatile organic compounds (VOCs) may be emitted from the furniture in the cabin [39] due to a rise in temperature (exposure to sunlight) and occupy the entire cabin, consequently increasing the overall concentration of VOCs within the cabin [40,41]. These VOCs are most likely to be hazardous to the health of drivers, who typically spend prolonged periods within the bus [42]. The VOC concentrations within the cabin change over time and vary with temperature. In other words, increased temperatures accelerate the dispersion of VOCs from the upholstery [40,42,43]. In the present study, the passenger zone of the bus generally maintained a temperature between 20 and 25°C during travel, which met the recommended air temperature (20–28°C) for Hong Kong bus cabins [40] and provided passengers a comfort space. Furthermore, based on the results of previous studies, temperatures measured in this study may have helped to decrease the dispersion of VOCs from bus furniture.

In summary, tour buses commissioned in the leisure travel in Taiwan have a low air exchange rate because most of time tour buses use AC system in recirculation mode, resulting in excessively high CO_2_ concentrations during high passenger capacity. The researchers observed CO_2_ concentrations double that suggested in Taiwan IAQ standards, which is a detriment to the safety of tourism transportation. Thus, the researchers suggest that tourism and transportation authorities and business owners pay greater attention to this problem. Furthermore, increasing frequency of air exchange in tour bus cabin during the journey is very beneficial to maintain the travel safety and enhance the quality of travel. Also, tour bus drivers are encouraged to open the bus doors and windows while the passengers disembark and the bus is on standby. Finally, although the tour buses monitored in the study maintained appropriate temperatures, the Ministry of Education has stipulated that tour buses commissioned for educational purposes must be of a manufacturing age of five years or less.
